# Textile Supplies for Hospitals

**Published:** 1916-03-04

**Authors:** 


### TEXTILE SUPPLIES FOR HOSPITALS

### VI.—Hospital Blankets.

The conditions seem to mark out blankets as supplies peculiarly capable of standardisation. The function of all bed blankets is one and the same, their treatment in use does not vary significantly, and they are intended for beds of standard and well-known dimensions. The circumstances might have been expected to produce a higher degree of uniformity than in point of fact exists. The public authorities who buy blankets, and who are by no means all hospital boards, pursue their individual ways without close reference to what others may be doing. The outcome is seen in a multiplicity of qualities and dimensions, all different and all intended to meet the same end. A certain width of variety is not essentially undesirable, but needless diversity leads to needless expense, first in manufacturing and next in holding in stock types that might reasonably be substituted one for another. The existence of individual eccentricities can be more easily stated than the reasons for them. What is known is that certain conventions were adopted in the course of a forgotten past, and are perpetuated from year to year as the custom of the establishment. In view of the extraordinary height of prices, many customary rules that are regarded as past changing might, at least, be reconsidered with an eye to any advantage there may be in using common standards.

### Wool, Woollen, and Union.

The equation of dimensions is a good deal simpler than the standardising of qualities, and is largely independent of it. The variety of practice is enough to suggest that a fair equality of value can be reached by various routes, and when different goods are purchased at different prices for similar purposes, it does not inevitably follow that either purchaser is at fault. The kinds of blanket available may be classified in different ways, and firstly in accordance with the material used in their manufacture. Ruling out the all-cotton blanket as definitely unsuitable for hospital requirements, three classes remain:—

(1) *All-wool* blankets, in the composition of which there is no other fibre than wool, be it wool straight from the sheep, or wool noils rejected by the wool comb.

(2) *Woollen* blankets, simulating all-wool, in which loose cotton and loose wool have been blended, carded, and spun together. The mixed yarn is oftenest in the warp, and when cotton is present there in appreciably more than 50 per cent., the warp behaves much like all-cotton yarn in burning.

(3) *Union blankets*, all-cotton in the warp direction, and all-wool in the weft. The cotton threads are very perceptibly finer than the wool, and serve the office simply of holding the wool constituent together.

The first-named are the most expensive and unquestionably the most desirable. The second-named are cheaper, and in a limited sense the presence of the cotton is advantageous, since in view of the inferior wool employed they would be less durable without cotton. The cheapest woollen blankets are, however, highly deceptive and to be avoided, as the saving in first cost is heavily out-balanced by the disparity in wear. The union blanket in its best forms and proportionately to its price is found to be not far inferior in point of value in use to similar blankets made from all-wool.

### Twill, Cloth, and Witney Blankets.

The classification in accordance with the components is put first, as it logically should be, but there are cross-divisions in accordance with the method of manufacture, and these are the ones that are first encountered commercially. Three types of blanket may be recognised:—

(*a*) The Scotch or Ayrshire blanket, named first not because of its absolute importance but because the type is a simple one, gradually extending in its employment. It is a twilled blanket, woven with a diagonal rib pattern, and in the original or "unraised" form it bears more resemblance to coarse cloth than to the better recognised types of blanket. In the original form it is more suitable for an under-blanket, to go next to the mattress, than for an over-blanket. Its warmth-giving, as apart from its wearing, properties are improved by raising a pile upon its surfaces.

(*b*) The cloth blanket, not twilled, but woven in plain basket-fashion, the threads crossing over and under alternately. This style of blanket is almost universally in household use in the Northern counties, and is coming into more extended use in the South. The type is used in public institutions in all parts of the country.

(*c*) The "raised" blanket, known as "Witney" when made in that Oxfordshire town and as "Yorkshire" blanket when made in that county. This blanket also is plain woven and differs from the cloth blanket in openness of structure and in the length of its pile.

Blankets are "raised" upon their surfaces in the interests of warmth, and are thereby made more bulky and more efficient containers of warmed air. The raising is done by means of drums covered with the spiny heads of the teazle plant, and of cylinders furnished with minor rollers toothed with flexible wire teeth. In either case the effect of the treatment is to detach fibre from the substructure of twisted yarn and to trail it upon the surface of the fabric. As may be readily comprehended, the raising is at the expense of the substructure and, if indefinitely continued, would tear the fabric entirely away. If no other consideration entered, the merits of the less-raised as against the highly-

Note.—Previous articles in this series appeared on December 18, 1915, and January 8 and 22, February 5 and 19, 1916. The next article will treat of "Hospital Clothing."

raised blanket would be beyond doubt or question, but the advantage in durability is also a matter of what, and of how, the blankets are made.

A cloth blanket is normally woven from finer-spun yarn than a raised blanket, and it is woven with more threads to the inch. Again, the cloth blanket is "milled" more heavily; milling being a process akin to the old-fashioned fulling or tucking, designed to consolidate the cloth by promoting the creeping of the fibres one upon another and the interlocking of the fine scales that are the distinctive feature of wool. Blankets meant to be raised more highly in course of finishing than the cloth variety are manufactured accordingly. They are made from longer fibre, more especially in the weft; they are spun from thicker yarn, often 50 per cent. thicker; they are woven into a looser structure, and are less closely milled since a proportion of fibres have to be extricated to form the face of the cloth. These differences between the raised and the cloth blanket can be appreciated in holding representative samples of both before the light. While the action of raising a pile subtracts from the durable value of any blanket, the injury is least in the case of those so made that they can best afford the subtraction, and is greatest in the cheapest qualities. Probably the worst value current in blankets lies in highly-raised cheap stuff made to attract the eye while new, and not constructed to wash and wear.

Blankets are not improved from the point of view of durability by the sulphur-stoving process through which they are put to whiten them, and the sulphur is more especially detrimental to those containing cotton. There are advantages in whiteness, but it is not actually indispensable to have blankets bleached. Manufacturers supply unbleached blankets which are cleaned simply with fuller's earth, raised gently while they are in a wet state, and bleached only by hanging in sunlight. The larger part of the blankets made and sold in these times are artificially dried upon the hot cylinders of a machine, but "summer-dried" and "sun-bleached" goods are obtainable, and these are blankets which have been dried upon stretching-frames in the open with, or without, sulphur bleaching. Goods so treated are perceptibly whiter than those dried by machine, and are intrinsically the better for avoiding an exposure to artificial heat. High temperatures denature and enfeeble wool, render it brittle on cooling, and destroy the scales upon the superficies of the fibre.

### Standard Specifications.

Blankets which part readily with fluff are undesirable for hospital use, both for the reason that dust is to be shunned, and because the fluff represents loss of weight which has been paid for, and loss also of warmth-giving power. A great deal is to be said for judging blankets not as they appear when new, but after they have been washed and dried a few times. In any event fixed standards may be applied to them, and for a standard size for 36 to 40 inch beds, the regular blanket of 66 by 86 inches, and weighing seven pounds a pair, is perhaps the most fitting. The standard of quantity is fixed by the conditions of manufacture. Blankets are divided into pairs for convenience in use, but initially they have to be woven in long, continuous pieces. A length of some 60 yards, or enough to cut into 25 pairs, is the blanket-maker's minimum quantity, and when less than 25 pairs are ordered, some portion of the original piece is left on hand. The economical way of buying blankets is to buy in multiples of 25 pairs, and as many multiples as the state of the market and the rate of consumption render advisable. The specifications of width, length, and weight present less difficulty than the accompanying factor which should be the crucial proof of the quality supplied. A complete specification ought to include two further details at least—the breaking strain and the elongation or elasticity. Tensile strengths may be improved by measures which are not actually to advantage in wear, because they involve greater bitterness. The strengths are tested by clamping a sample of given size between the vice-like jaws of a machine, and applying strain by the turning of a screw-handle. The stress at which the fabric breaks is read upon a dial, and the extent to which the sample stretches before breaking is noted at the same time. A standard is best arrived at by the comparison of a variety of patterns of acknowledged suitability, and the standard adopted should in any case not be fixed at the top notch, but at such a reasonable lower height as can be reached by the manufacturer consistently. Atmospheric conditions alone suffice to cause a sample to resist more pull upon one day than another, and the inevitable minor incidents of manufacture introduce differences that are at all events perceptible.

A standard specification is incomplete without a sample to show the quality of material and the style of make, and, indeed, it is no more than a statement of the minimum requirement in certain named directions. It is the complement to a sample, and meant to secure that the qualities of the original shall be adhered to fully. An insensible degradation of standards goes on when the deliveries of one year are made the standards for the next. Without the knowledge of the purchasing authority the standard becomes progressively more debased, and even if the error of changing the official sample be avoided, it is a practical impossibility for unpractised judges to check the deviations of quality which a testing machine will reveal. The eye can tell when the threads per inch are reduced in number, but it is not so easy to determine whether weak materials have been substituted for strong. An expert may have his suspicions, but it is the machine which vouches for their truth or error by putting the resultants to the test. Standard specifications have been formulated for the blankets purchased by the Army authorities, and the millions of blankets that have been made with satisfaction to both sides is some evidence that a standard could be set up for hospital use to serve as a protection to the purchaser and to the manufacturer.

